# Factors associated with the duration of telephone observation and consultation sessions provided by the Hiroshima Prefecture Follow-up Center in the later stages of the COVID-19 pandemic in Japan

**DOI:** 10.1371/journal.pone.0352251

**Published:** 2026-06-26

**Authors:** Yui Yumiya, Mikikazu Itakura, Noriyuki Shiroma, Hanako Murayama, Chiaki Taguchi, Toki Yamashita, Chiaki Tokubo, Takahito Yoshida, Tatsuhiro Nagata, Odgerel Chimed-Ochir, Ami Fukunaga, Kanako Kitahara, Hiroki Ohge, Masao Kuwabara, Tatsuhiko Kubo

**Affiliations:** 1 Department of Public Health and Health Policy, Graduate School of Biomedical and Health Sciences, Hiroshima University, Hiroshima, Japan; 2 School of Medicine, Hiroshima University, Hiroshima, Japan; 3 Hiroshima Prefectural Eastern Health Center, Hiroshima, Japan; 4 Hiroshima Prefectural Government Health and Welfare Affairs Bureau, Hiroshima, Japan; 5 Department of Infectious Diseases, Hiroshima University Hospital, Hiroshima, Japan; 6 Hiroshima Prefectural Center for Disease Control and Prevention, Hiroshima, Japan; Universita Cattolica del Sacro Cuore Sede di Roma, ITALY

## Abstract

**Background:**

Identifying the factors that affected the workload of Public Health Centers (PHCs) during the recent COVID-19 pandemic may help such institutions improve their preparedness and response to future pandemics and public health crises. Therefore, this study aimed to examine factors related to the duration of telephone observation and consultation sessions administered by nurses at the Hiroshima Prefecture Follow-Up Center to patients with COVID-19 recuperating at home during the pandemic.

**Methods:**

This cross-sectional study used data from 22,490 telephone observation and consultation sessions collected using the COVID-19 Japan-Surveillance in Post-Extreme Emergencies and Disasters form by nurses at the Hiroshima Prefecture Follow-Up Center (Japan) from 1 November 2022 to7 May 2023. Long call duration was defined as ≥ 15 minutes. Study days were classified into low-, middle-, and high-incidence groups using the 25th and 75th percentiles of the 7-day moving average of newly diagnosed COVID-19 cases. Multivariable logistic regression models were fitted separately for each incidence group.

**Results:**

Across the low-, middle-, and high-incidence groups, age ≥ 80 years, two or more symptoms, medical consultation on physical symptoms, and consultation about family members or close contacts were consistently associated with long telephone observation and consultation sessions. In the middle- and high-incidence groups, long telephone sessions were significantly more common at nighttime and among patients who requested physician involvement, and less common among pregnant women, whereas longer evening sessions were observed only in the high-incidence group.

**Conclusions:**

In the later stages of the COVID-19 pandemic, several factors found to be associated with long telephone observation and consultation sessions depended on the number of newly diagnosed cases. These results may help PHCs and Follow-Up Centers plan staffing, triage, and workflows when personnel and time are limited.

## Introduction

The rapid spread of COVID-19 during the recent global pandemic strained the resources of many public health centers (PHCs) and led to problems that affected patient admissions and collaborations with related agencies and institutions [1,2]. In more typical times, PHCs in Japan provide a wide range of services, including emergency management, the collection of vital statistics, and the provision of services related to nutrition and food sanitation, hygiene and public health, the health of children, mothers, and older adults, dental health, mental health, care and social support for patients with rare diseases, and the prevention of infectious diseases (e.g., HIV/AIDS, tuberculosis, sexually transmitted diseases) [3]. However, during the COVID-19 pandemic, there were insufficient numbers of PHCs and public health nurses, which rapidly increased workloads beyond the capacity of these institutions, thereby necessitating the transfer or outsourcing of many health-care services to other facilities so that the PHCs could continue to function [4]. Hiroshima Prefecture established a follow-up center in December 2021 to provide health-related observation and consultation sessions to patients with COVID-19 who were recuperating at home [5]. This initiative was essential in alleviating the workload of PHCs. However, health consultations often require careful attention, which can take time, thereby depleting limited resources such as time and staff availability and potentially causing delays in care for other patients. Therefore, it is essential to evaluate the effectiveness of these services and crisis management strategies, particularly in terms of the temporal efficiency of outsourcing operations and task shifting, to lay the foundation for implementing more effective and efficient interventions. However, to our knowledge, few studies have performed quantitative evaluations of healthcare activities during these types of emergencies [6].

Hiroshima Prefecture collected data from telephone observations and consultation sessions that were provided by nurses at its follow-up center from 23 December 2021 to 7 May 2023, using the Hiroshima Prefecture COVID-19 version of the Japan-Surveillance in Post-Extreme Emergencies and Disasters (COVID-19 J-SPEED) form [7]. The objective of the present study was to identify factors associated with the duration of telephone observation and consultation sessions provided by nurses at this follow-up center to patients with COVID-19 who were recuperating at home. Our findings may help identify areas for potential improvement, thereby providing a foundation for enhancing operational efficiency and developing more effective methods for delivering public health services.

## Materials and methods

### Study design and data collection

This cross-sectional study used the COVID-19 J-SPEED form to collect data [7]. The original J-SPEED form was designed to simplify and standardize the collection of health data for situations requiring near real-time responses, and was developed in response to the 2011 Great East Japan Earthquake [8]. The version of the tool used in the present study was modified in that it considered hospitals [9], centers that performed polymerase chain reaction (PCR) testing [10,11], centers that administered online medical treatments [12], PHCs [13], recovery accommodation facilities, follow-up centers for patients recuperating at home, and centers that provided oxygen therapy. In this study, all data were obtained from patients who were recuperating at home from COVID-19 and received telephone observations and/or consultations from nurses at the Hiroshima Prefecture Follow-Up Center [7].

**Fig 1:**
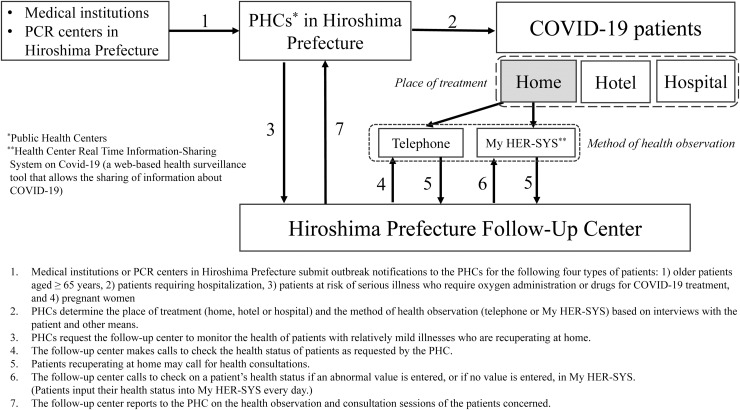
Roles of the Hiroshima Prefecture Follow-Up Center.

The Hiroshima Prefecture Follow-Up Center was open 24 hours per day/7 days per week, and had three main roles: (*i*) observing patients who were recuperating from COVID-19 while at home by telephone or My HER-SYS (Health Center Real Time Information-Sharing System on COVID-19) [14,15]; (*ii*) administering health consultations to patients; and (*iii*) reporting patient responses to the PHC in Hiroshima Prefecture as necessary (Fig 1).

The patients in this study met all of the following four inclusion criteria: 1) had visited a medical institution or PCR center in Hiroshima Prefecture and tested positive for COVID-19; 2) were legally eligible for outbreak notification, excluding those in Hiroshima, Kure, and Fukuyama cities; 3) were recuperating from COVID-19 at home with health observations by telephone or My HER-SYS; and 4) were recuperating from COVID-19 at home and seeking telephone-based health consultation. The following four types of patients were legally eligible for outbreak notifications: 1) older patients aged ≥ 65 years, 2) patients requiring hospitalization, 3) patients at risk of serious illness who required oxygen administration or drugs for COVID-19 treatment, and 4) pregnant women [16]. Among these four types of patients recuperating from COVID-19 at home, the PHCs managed patients with severe conditions or who were expected to require hospitalization. For the other patients, the follow-up center provided health observation and/or consultation sessions by telephone or My HER-SYS [7]. The three roles of the follow-up center (described above) were in accordance with the operations manual prepared by the Hiroshima Prefectural Government for the Hiroshima Prefecture Follow-Up Center. The health observation methods to be used were determined by the PHCs through interviews with patients and other means, considering the patient's age and ability to use My HER-SYS. However, for patients aged ≥ 65 years, it was decided that health observations would be conducted by telephone, regardless of the patient's ability to use the online system.

The COVID-19 J-SPEED form used by the follow-up center, which is composed of 48 items, was used to record demographic data, initial contact route, patient symptoms, consultation details, responses of the nurse, outcomes of medical coordination after the nurse response, and the duration of the telephone observation and consultation session [16–20]. From 23 December 2021 to 7 May 2023, registered nurses participated in 89,434 telephone calls to observe and consult with patients with COVID-19 at home. As circumstances changed during this period, however, the national government and Hiroshima Prefecture changed the criteria for both hospitalization and outbreak notifications. Consequently, all data in the present study were from 1 November 2022 to 7 May 2023 (the later stages of COVID-19), when these criteria remained the same. This study therefore analyzed 22,490 telephone observation and consultation sessions provided by nurses at the Hiroshima Prefecture Follow-Up Center.

### Data analysis

The variables related to call duration were selected based on a review of prior research [17–21] and interviews with frontline staff. The final set of predictors comprised age group, sex/pregnancy category, contact time, number of symptoms, and consultation characteristics. Age was categorized as 0–64, 65–79, and ≥ 80 years, with 65–79 years used as the reference category because it represented the largest group. Sex/pregnancy category was classified as non-pregnant female, male, and pregnant female; non-pregnant woman was used as the reference category because it represented the largest group and served as the standard comparison category for evaluating differences associated with male sex or pregnancy. Contact time was categorized as “daytime” (08:30–17:15), “evening time” (17:15–20:00) or “nighttime” (20:00–08:30), with daytime used as the reference category because it was the most frequent contact period. Symptoms included fever (≥ 37.5°C), oxygen saturation ≤ 95%, respiratory symptoms (e.g., dyspnea, cough, sore throat), fatigue, digestive symptoms (e.g., vomiting, diarrhea), loss of smell or taste, poor diet or fluid intake, stress-related symptoms, need for emergency mental care, and other symptoms. The number of symptoms was categorized as 0, 1, 2, and ≥ 3 symptoms and included in the regression models as a categorical variable, with 0 symptoms used as the reference category. Consultation characteristics included medical consultation on physical symptoms, request for a physician's involvement, concerns about the patient's own life (e.g., financial concerns, need to stay at home), concerns about family and close contacts (e.g., health and infection prevention measures), and others.

The primary outcome, call duration, was originally recorded as an ordinal variable with three categories: < 15 minutes, 15–30 minutes, and ≥ 30 minutes. In this study, long call duration was defined as ≥ 15 minutes. This definition was based on the operational threshold used in the original workflow and prior evidence indicating that the average call duration was less than 15 minutes [21].

Of 22,982 case records during the study period, 22,490 records had non-missing incidence-group classification and call-duration outcome and were included in the analyses. After excluding records with missing covariates, 22,372 records were included in the regression analyses. For the primary analysis, incidence groups were defined using the 25th and 75th percentiles of the 7-day moving averages (MAs) of newly diagnosed COVID-19 cases during the study period. The 7-day MA was selected to reduce short-term day-to-day fluctuations, including weekday effects, variation in testing and reporting volume, and delays between diagnosis and the initiation of telephone observation or consultation [22]. This approach was appropriate for classifying broader incidence periods rather than capturing transient daily changes. The 25th and 75th percentile cutoffs were 281.3 and 3021.0 cases, respectively. Days with a 7-day MA at or below 281.3 cases were classified as the low-incidence group, days with values above 281.3 and at or below 3021.0 cases as the middle-incidence group, and days with values above 3021.0 cases as the high-incidence group. Thus, days at the percentile thresholds were assigned to the lower adjacent group. The observed ranges of the 7-day MA were 200.7–281.3 cases in the low-incidence group, 281.4–2975.9 cases in the middle-incidence group, and 3036.0–6248.0 cases in the high-incidence group. This resulted in 46 low-incidence days, 90 middle-incidence days, and 46 high-incidence days. In the descriptive analysis, these groups included 1,659, 8,959, and 11,872 telephone observation and consultation sessions, respectively. Multivariable binary logistic regression models were fitted separately for each incidence group to estimate adjusted odds ratios (aORs) and 95% confidence intervals (CIs). Multicollinearity was assessed using the variance inflation factor (VIF) in each incidence group-specific primary model. All VIF values were below 2. Model discrimination and calibration were assessed using the area under the receiver operating characteristic curve (AUC), Brier score, and Hosmer–Lemeshow test.

Several sensitivity and supplementary analyses were conducted. First, to evaluate the robustness of the incidence-group classification, analyses were repeated using four alternative definitions: tertile-based grouping of the 7-day MA, grouping based on raw daily case counts using the 25th and 75th percentiles, and grouping based on 3-day and 10-day MAs using the 25th and 75th percentiles. The tertile cutoffs for the 7-day MA were 318.1 and 2659.2 cases. The 25th and 75th percentile cutoffs were 302.5 and 2977.3 cases for raw daily case counts, 293.6 and 3101.3 cases for the 3-day MA, and 278.8 and 3164.8 cases for the 10-day MA. These values represent the cutoffs used to define the incidence groups and therefore fall between the observed ranges of adjacent groups; the observed ranges under each definition are shown in S1 Table. Second, ordinal logistic regression was conducted using the original three-level call-duration outcome to assess whether dichotomizing call duration affected the findings (S2 Table). Third, variables related to initial contact route/workflow and outcomes of medical coordination after nurse response were summarized but not included in the primary regression models (S3 Table). Initial contact route/workflow variables included end-of-isolation contact, My HER-SYS-related contact, initial contact from the follow-up center, direct calls from patient or family members, and others. My HER-SYS-related contacts included calls triggered by abnormal values detected through My HER-SYS or by missing/unregistered entries. These variables were excluded from the primary models because they represented heterogeneous operational pathways and could partly reflect consultation complexity emerging during the call rather than independent patient-level clinical characteristics. Outcomes of medical coordination after nurse response, including end-of-isolation contact, hospitalized, coordinated for hospitalization but could not be admitted, transferred response from the follow-up center to a PHC, ongoing (coordinating for hospitalization, or contacting a PHC), and others were also excluded because they represented downstream processes occurring during or after nurse assessment, and several categories were sparse or absent. Fourth, the distribution of individual symptoms within each symptom-count category was summarized to characterize the composition of the symptom-count variable (S4 Table). Finally, pregnancy-related subgroup and exploratory interaction analyses were conducted to provide more detailed information on the sex/pregnancy category and to examine potential heterogeneity in associations with long call duration. Using the regression dataset after excluding records with missing covariates, we summarized the number of pregnant callers and long call duration by incidence group. We also fitted exploratory logistic regression models with interaction terms for sex/pregnancy category × contact time and sex/pregnancy category × number of symptoms. These analyses were considered exploratory because the number of long calls among pregnant callers was small. All analyses were performed using R version 4.5.1. The R packages used were readxl (1.5.0), dplyr (1.2.1), tidyr (1.3.2), tibble (3.3.1), broom (1.0.13), car (3.1.5), pROC (1.19.0.1), ResourceSelection (0.3.6), writexl (1.5.4), and MASS (7.3.65). All data were anonymized to ensure confidentiality.

Approval for ethical review was obtained from Hiroshima University on 29 June 2021 (approval No.: E-2508). All data are anonymized, no patient-identifiable data were recorded at any time, and informed consent of the patients was not required. The Ethics Committee of Hiroshima University approved the waiver of informed consent.

This study was supported by a grant from the Japan Agency for Medical Research and Development (grant numbers: JP20fk0108453h0001, JP21fk0108550h0001) and JSPS KAKENHI Grant Number (JP23K16256).

## Results

**Fig 2:**
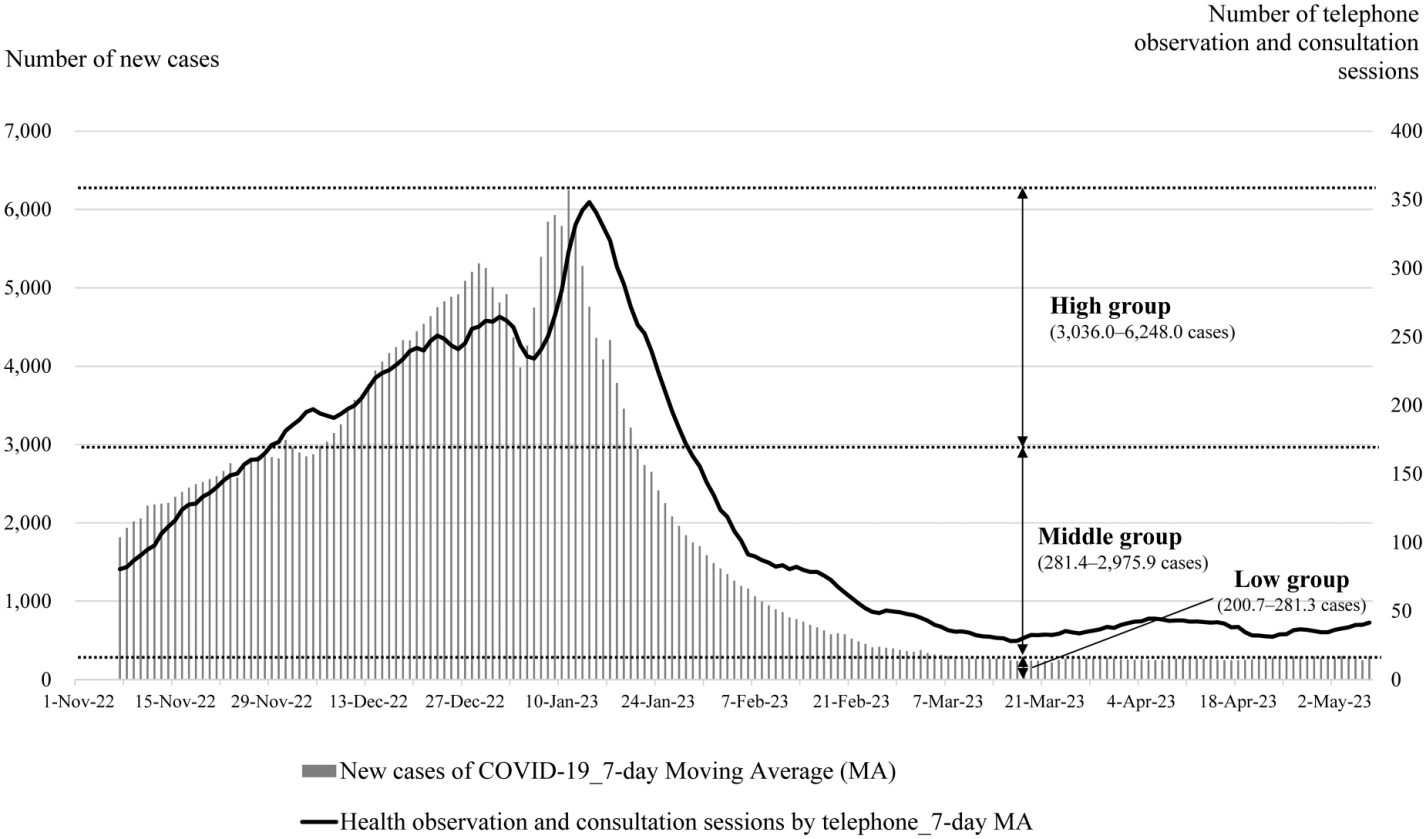
Seven-day moving average (MA) numbers of new cases of COVID-19 and telephone observation and consultation sessions at the Hiroshima Prefecture Follow-Up Center in the later stages of the COVID-19 pandemic. The bars represent the 7-day MA number of newly diagnosed COVID-19 cases, and the solid line represents the 7-day MA number of telephone observation and consultation sessions. The dotted horizontal lines indicate the 25th and 75th percentile cutoffs used to define incidence groups. Values in parentheses indicate the observed ranges of the 7-day MA within each group.

The registered nurses at the follow-up center provided 22,490 telephone observation and consultation sessions in the later stages of the COVID-19 pandemic. The chronological patterns of changes in the 7-day MA number of new COVID-19 cases and telephone observation and consultation sessions were similar during this period (Fig 2).

**Fig 3:**
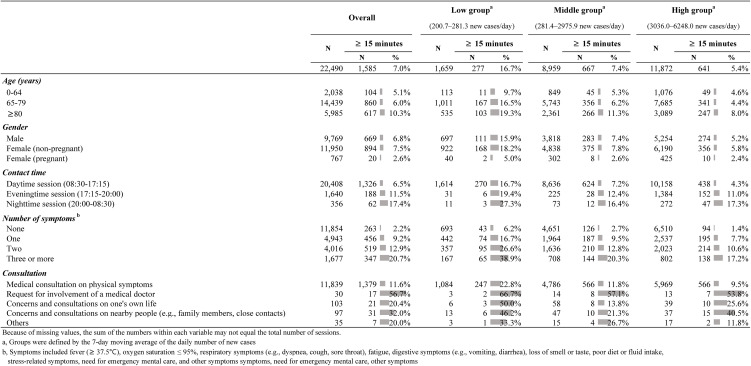
Characteristics of long telephone observation and consultation sessions (≥ 15 minutes) in three groups defined by the 7-day moving average (MA) of newly diagnosed COVID-19 cases during the later stage of the pandemic.

Among all 22,490 telephone observation and consultation sessions, 1,585 (7.0%) lasted ≥ 15 minutes (Fig 3). The proportion of long sessions was highest in the low-incidence group (16.7%), followed by the middle-incidence group (7.4%) and the high-incidence group (5.4%). Overall, long sessions were more frequent among individuals aged ≥ 80 years (10.3%), non-pregnant female patients (7.5%), nighttime sessions (17.4%), and patients requesting physician involvement (56.7%). The proportion of long sessions increased with symptom burden, from 2.2% among patients with no symptoms to 20.7% among those with three or more symptoms. However, the proportions varied across incidence groups for nighttime sessions, concerns about the patient's own life, and concerns about family members or close contacts.

We then analyzed these data using multivariable binary logistic regression analysis and calculated adjusted odds ratios (aORs; Table 1). The number of symptoms was consistently associated with long telephone observation and consultation sessions across all incidence groups. Compared with patients with no symptoms, those with a higher number of symptoms had progressively higher odds of long telephone observation and consultation sessions across all incidence groups. This pattern was particularly clear for patients with two symptoms and those with three or more symptoms, with the latter showing the highest odds in all incidence groups (low: aOR = 6.07, 95% CI: 3.28–11.22; middle: aOR = 5.04, 95% CI: 3.40–7.47; high: aOR = 6.09, 95% CI: 4.02–9.22). Medical consultation on physical symptoms was also associated with long sessions in all incidence groups (low-incidence group: aOR = 2.23, 95% CI: 1.20–4.16; middle-incidence group: aOR = 2.14, 95% CI: 1.46–3.15; high-incidence group: aOR = 2.57, 95% CI: 1.70–3.89). Concerns or consultations regarding family members or close contacts were likewise associated with long sessions in the low-incidence group (aOR = 4.15, 95% CI: 1.25–13.83), middle-incidence group (aOR = 2.20, 95% CI: 1.01–4.82), and high-incidence group (aOR = 5.78, 95% CI: 2.72–12.30). Patients aged ≥ 80 years had higher odds of long sessions (low-incidence group: aOR = 1.34, 95% CI: 1.00–1.80; middle-incidence group: aOR = 1.80, 95% CI: 1.51–2.14; high-incidence group: aOR = 1.67, 95% CI: 1.39–1.99).

**Table 1:** Factors associated with long telephone observation and consultation sessions (≥ 15 minutes) by incidence group.

	Low group^a^ (200.7–281.3 new cases/day)			Middle group^a^ (281.4–2975.9 new cases/day)			High group^a^ (3036.0–6248.0 new cases/day)		
	aOR^b^	95%CI	*P-*value^c^	aOR^b^	95%CI	*P-*value^c^	aOR^b^	95%CI	*P-*value^c^
*Age (years)*									
65–79	ref			ref			ref		
0–64	0.54	0.24–1.23	0.144	0.82	0.57–1.17	0.266	0.95	0.67–1.36	0.785
≥ 80	**1.34**	**1.00–1.80**	**0.049**	**1.80**	**1.51–2.14**	**<0.001**	**1.67**	**1.39–1.99**	**<0.001**
*Sex/pregnancy category*									
Female (non-pregnant)	ref			ref			ref		
Male	0.96	0.72–1.27	0.760	0.98	0.83–1.16	0.856	0.89	0.75–1.06	0.199
Female (pregnant)	0.36	0.07–1.91	0.228	**0.37**	**0.17–0.81**	**0.013**	**0.37**	**0.18–0.74**	**0.005**
*Contact time*									
Daytime session (08:30–17:15)	ref			ref			ref		
Evening session (17:15–20:00)	1.37	0.55–3.44	0.496	1.06	0.69–1.61	0.792	**1.38**	**1.12–1.69**	**0.002**
Nighttime session (20:00–08:30)	2.83	0.60–13.36	0.188	**2.03**	**1.04–3.93**	**0.037**	**2.75**	**1.94–3.91**	**<0.001**
*Number of symptoms* ^*d*^									
None	ref			ref			ref		
One	1.76	0.99–3.13	0.052	**1.93**	**1.33–2.79**	**<0.001**	**2.31**	**1.56–3.43**	**<0.001**
Two	**3.19**	**1.81–5.63**	**<0.001**	**2.82**	**1.94–4.10**	**<0.001**	**3.43**	**2.31–5.10**	**<0.001**
Three or more	**6.07**	**3.28–11.22**	**<0.001**	**5.04**	**3.40–7.47**	**<0.001**	**6.09**	**4.02–9.22**	**<0.001**
*Consultation*									
Medical consultation on physical symptoms	**2.23**	**1.20–4.16**	**0.011**	**2.14**	**1.46–3.15**	**<0.001**	**2.57**	**1.70–3.89**	**<0.001**
Request for involvement of a medical doctor	2.27	0.17–29.84	0.532	**5.75**	**1.88–17.55**	**0.002**	**8.84**	**2.75–28.45**	**<0.001**
Concerns and consultations on one’s own life	3.66	0.67–20.06	0.135	0.92	0.41–2.07	0.842	2.23	0.96–5.19	0.064
Concerns and consultations on nearby people (e.g., family members, close contacts)	**4.15**	**1.25–13.83**	**0.020**	**2.20**	**1.01–4.82**	**0.048**	**5.78**	**2.72–12.30**	**<0.001**

Abbreviations: aOR: adjusted odds ratio, 95%CI: 95% confidence interval.

^a^Incidence groups were defined using the 25th and 75th percentiles of the 7-day moving average of newly diagnosed COVID-19 cases.

^b^aOR > 1: session lasting ≥ 15 minutes.

^c^Binary logistic regression.

^d^Symptoms included fever (≥ 37.5°C), oxygen saturation ≤ 95%, respiratory symptoms (e.g., dyspnea, cough, sore throat), fatigue, digestive symptoms (e.g., vomiting, diarrhea), loss of smell or taste, poor diet or fluid intake, stress-related symptoms, need for emergency mental care, and other symptoms.

Nighttime sessions were associated with long sessions in the middle- and high-incidence groups (middle-incidence group: aOR = 2.03, 95% CI: 1.04–3.93; high-incidence group: aOR = 2.75, 95% CI: 1.94–3.91). Evening sessions were associated with long sessions only in the high-incidence group (aOR = 1.38, 95% CI: 1.12–1.69). Requests for physician involvement were strongly associated with long sessions in the middle-incidence group (aOR = 5.75, 95% CI: 1.88–17.55) and high-incidence group (aOR = 8.84, 95% CI: 2.75–28.45). By contrast, pregnant female had lower odds of long sessions in the middle-incidence group (aOR = 0.37, 95% CI: 0.17–0.81) and high-incidence group (aOR = 0.37, 95% CI: 0.18–0.74).

Sensitivity analyses generally supported the robustness of the primary findings. Results were broadly consistent when alternative incidence-group definitions were used, including tertile-based grouping of the 7-day MA, raw daily case counts, and 3-day and 10-day MAs (S1 Table). Ordinal logistic regression using the original three-level call-duration outcome (<15, 15–30, and ≥ 30 minutes) also showed patterns similar to those of the primary binary logistic regression analysis (S2 Table). The number of symptoms, medical consultation on physical symptoms, and age ≥ 80 years were consistently associated with longer call-duration categories across all incidence groups. The primary binary logistic regression models showed acceptable discrimination and calibration. The AUCs were 0.741, 0.742, and 0.781, and the Brier scores were 0.1242, 0.0644, and 0.0471 for the low-, middle-, and high-incidence groups, respectively. Hosmer–Lemeshow tests did not indicate clear evidence of poor calibration.

Among pregnant callers in the regression dataset, 40, 301, and 418 sessions were classified into the low-, middle-, and high-incidence groups, respectively, with 2 (5.0%), 8 (2.7%), and 10 (2.4%) long sessions. Exploratory interaction analyses showed no clear evidence that the association between symptom burden and long call duration differed by sex/pregnancy category (*p* = 0.643), while the interaction between sex/pregnancy category and contact time was borderline (*p* = 0.067).

Initial contact route/workflow and outcomes of medical coordination after nurse response are summarized in S3 Table. Long-session proportions varied across initial contact route/workflow categories, ranging from 2.0% for end-of-isolation contacts to 22.6% for direct calls from patients or family members. Several medical coordination outcome categories were sparse or absent, including hospitalization, transfer to a PHC, ongoing coordination, and coordinated hospitalization without admission.

## Discussion

In the present study, we examined factors associated with long telephone observation and consultation sessions among patients with COVID-19 recuperating at home during the later stage of the pandemic in Hiroshima Prefecture. Across incidence groups, the number of symptoms, medical consultation on physical symptoms, age ≥ 80 years and consultation about family members or close contacts were consistently associated with long call duration. During middle- and high-incidence periods, long calls were more common among patients with nighttime contacts, and those requesting physician involvement, whereas pregnant callers had lower odds of long call duration.

### Factors related to COVID-19 infection

Across all three incidence groups, long call duration was consistently associated with higher numbers of symptoms, medical consultation on physical symptoms, and age ≥ 80 years, even after adjusting for potential confounding. Compared with in-person consultation, telephone triage can be more complex because nurses cannot rely on nonverbal cues and visual assessment [23,24], which may affect a patient's ability to fully communicate symptoms [25,26]. Hiroshima Prefecture established criteria for problematic physical symptoms that the follow-up center should consider before reporting to the PHCs and related agencies. Therefore, patients with a higher number of symptoms or those requesting consultation regarding physical symptoms may have required more time for nurses to assess whether they met these criteria [27].

For patients aged ≥ 80 years, consultation time was likely longer [19–21,28] because telephone assessment can be more difficult when patients have hearing difficulties [29], cognitive decline [30], lower health literacy [31], or difficulty using medical devices. For example, Hiroshima Prefecture lent pulse oximeters free of charge to patients recuperating at home [32], and the follow-up center may have needed time to explain their operation to patients or confirm oxygen saturation values. Because older patients tend to have poorer blood flow and may need more time to learn how to use a medical device [33], they may have required more time for SpO_2_ measurement.

Likewise, the duration of the telephone session was longer in all three groups when a patient had concerns and requested consultation on health and infection prevention measures related to family and close contacts. The consistent association across all incidence groups may reflect a persistently high level of fear regarding the infection of family and close contacts, which is in line with the official report of the Ministry of Health, Labour and Welfare in Japan [34], showing that concern about infection remained above 40% throughout the pandemic. In terms of the duration of a consultation session, previous studies in general practice settings reported that longer consultation times were associated with provision of preventive health care [17] and mental health care [18,19]. Thus, the longer sessions provided by the Follow-Up Center may have helped to reduce patient fears by explaining specific measures to be used for preventing the infection of others.

### Factors related to incidence level and operational context

Although the overall proportion of long sessions decreased from the low- to high-incidence groups, several factors were associated with long sessions during middle- and high-incidence periods. Nighttime contacts were associated with long sessions in the middle- and high-incidence groups, and evening contacts were associated with long sessions in the high-incidence group. One possible explanation is that patients may have experienced worsening symptoms or increased anxiety outside regular clinic hours, when access to family physicians was limited. In addition, previous studies have shown that mental health consultations tend to require more time than other types of consultations [18,19]. This interpretation is also supported by a previous Irish crisis helpline study during the COVID-19 lockdown, which reported that average call duration increased overall and that the longest average call durations were observed in the early morning hours [35].

Requests for physician involvement were strongly associated with long sessions in the middle- and high-incidence groups. These sessions may have required additional time because nurses needed to assess clinical urgency, determine whether physician involvement was necessary, and coordinate subsequent care. The PHCs in this study area served mountainous regions of Japan, where access to medical facilities may have been limited. During periods of rapidly increasing COVID-19 cases, hospital beds for COVID-19 patients were reportedly under pressure or fully occupied [36], which may have increased the need for careful telephone assessment and coordination by the Follow-Up Center. In addition, some patients may have sought physician involvement through telephone or online consultation when contacting their family physician was difficult [36–39]. However, this interpretation should be made cautiously because the number of sessions involving requests for physician involvement was small.

Pregnant women had lower odds of long sessions in the middle- and high-incidence groups. One possible explanation is that pregnant women managed by the Follow-Up Center may have had relatively mild illness, because pregnant women in late pregnancy or those at high risk were more likely to be followed by PHCs. In addition, pregnant women were encouraged to consult their family obstetric clinic or unit if symptoms worsened [37]. However, this finding should be interpreted cautiously because the number of long sessions among pregnant callers was small. Moreover, the dataset did not include detailed obstetric risk status, gestational age, or subsequent consultation with obstetric services; therefore, the reasons for shorter sessions among pregnant women could not be directly confirmed.

Our findings suggest that identifying factors associated with longer calls may help PHCs and follow-up centers plan staffing, triage, and workflows when personnel and time are limited. Remote consultation systems and user-friendly symptom-reporting tools may also support home-based care for older adults in future health crises, although their implementation should consider digital literacy barriers and the need for support from family members, caregivers, and trained staff [38–41].

This study has several limitations. First, not all patients with COVID-19 recuperating at home who met the inclusion criteria were included, because patients anticipated to require hospitalization were monitored directly by PHCs. The J-SPEED data also did not include health observation conducted only through My HER-SYS. These factors may limit the representativeness and generalizability of the findings. Second, this study was conducted in the later stages of the COVID-19 pandemic in Japan, after major policy changes including the shift toward a “With-Corona” approach, narrowing of outbreak notification criteria, and shortening of isolation periods [16,42]. Although the analysis was restricted to a period when outbreak notification and hospitalization criteria were relatively stable, public perception, anxiety, and consultation needs may have changed during the study period, particularly in its early phase, and may have acted as drivers of longer calls. Third, call duration may have been influenced by operational demands. During high-incidence periods, nurses may have needed to assess more patients per unit time, potentially shortening individual calls. Although staffing was adjusted operationally according to service demand, detailed staffing data were not available for inclusion in the models. Fourth, initial contact route/workflow and outcomes of medical coordination after nurse response were not included in the primary models. Although these variables were summarized in supplementary analyses, they may partly reflect case-mix and operational complexity. Their exclusion may therefore have contributed to residual confounding. Fifth, the present analysis focused on the number of symptoms rather than specific symptom types; identifying which particular symptoms are associated with longer consultations may further refine triage protocols and represents an important direction for future research. Finally, available demographic and social-context variables were limited. Although age and sex/pregnancy category were included, the dataset did not contain information on caregiver role, household composition, language support, socioeconomic status, nationality, patient origin, or the number of calls per patient. Future surveillance systems should consider incorporating these variables while ensuring data privacy.

## Conclusions

Among patients with COVID-19 recuperating at home in Hiroshima Prefecture during the later stage of the pandemic, a greater number of symptoms, medical consultation on physical symptoms, age ≥ 80 years, and consultation about family members or close contacts were consistently associated with longer telephone observation and consultation sessions. Nighttime contacts and requests for physician involvement were associated with longer sessions mainly during middle- and high-incidence periods, whereas pregnancy was associated with lower odds of long sessions and should be interpreted cautiously because the number of long sessions among pregnant callers was small. These findings may help PHCs and follow-up centers anticipate consultations requiring more time and inform staffing, triage, and workflow planning under limited personnel and time. Remote consultation systems and user-friendly symptom-reporting tools may also support home-based care for older adults in future health crises.

## Supporting information

S1 Table Factors associated with long telephone observation and consultation sessions under four alternative incidence-group definitions. This file contains four tables based on alternative definitions of the incidence groups: tertiles of the 7-day moving average (S1A), the 25th and 75th percentiles of raw daily case counts (S1B), the 25th and 75th percentiles of the 3-day moving average (S1C), and the 25th and 75th percentiles of the 10-day moving average (S1D).
(DOCX)

S2 Table Ordinal logistic regression analysis of telephone observation and consultation duration (<15, 15–30, and ≥30 minutes). (DOCX)

S3 Table Initial contact route/workflow and outcomes of medical coordination after nurse response by call duration. (DOCX)

S4 Table Distribution of individual symptoms by symptom-count category. (DOCX)

## Abbreviations

J-SPEED: Japan-Surveillance in Post-Extreme Emergencies and Disasters
MA: Moving Average
PHC: Public Health Center
