# Supplementary material for: Factors associated with the duration of telephone observation and consultation sessions provided by the Hiroshima Prefecture Follow-up Center in the later stages of the COVID-19 pandemic in Japan
Source: PLoS One. 2026 Jun 26;21(6):e0352251. doi: 10.1371/journal.pone.0352251 (PMC13308847; doi:10.1371/journal.pone.0352251)
Supplement: S1 Table — This file contains four tables based on alternative definitions of the incidence groups: tertiles of the 7-day moving average (S1A), the 25th and 75th percentiles of raw daily case counts (S1B), the 25th and 75th percentiles of the 3-day moving average (S1C), and the 25th and 75th percentiles of the 10-day moving average (S1D). (DOCX) [file pone.0352251.s001.docx]

**S1A Table. Factors associated with long telephone observation and consultation sessions under four alternative incidence-group definitions (tertiles of the 7-day moving average)**

|  | **Low group^a^**  **(200.7–315.9 new cases/day)** | | |  | **Middle group^a^**  **(322.6–2652.6 new cases/day)** | | |  | **High group^a^**  **(2662.6–6248.0 new cases/day)** | | |
| --- | --- | --- | --- | --- | --- | --- | --- | --- | --- | --- | --- |
|  | **aOR^b^** | **95%CI** | ***P-*value^c^** |  | **aOR^b^** | **95%CI** | ***P-*value^c^** |  | **aOR^b^** | **95%CI** | ***P-*value^c^** |
| *Age (years)* |  |  |  |  |  |  |  |  |  |  |  |
| 65–79 | ref |  |  |  | ref |  |  |  | ref |  |  |
| 0–64 | 0.93 | 0.51–1.67 | 0.802 |  | 0.84 | 0.54–1.30 | 0.430 |  | 0.82 | 0.59–1.15 | 0.251 |
| ≥ 80 | **1.56** | **1.21–2.01** | **<0.001** |  | **1.82** | **1.47–2.26** | **<0.001** |  | **1.64** | **1.39–1.92** | **<0.001** |
| *Sex/pregnancy category* |  |  |  |  |  |  |  |  |  |  |  |
| Female (non- pregnant) | ref |  |  |  | ref |  |  |  | ref |  |  |
| Male | 0.93 | 0.73–1.19 | 0.569 |  | 1.03 | 0.83–1.26 | 0.809 |  | 0.91 | 0.78–1.06 | 0.219 |
| Female (pregnant) | **0.22** | **0.06–0.82** | **0.025** |  | 0.52 | 0.22–1.24 | 0.139 |  | **0.36** | **0.18–0.71** | **0.003** |
| *Contact time* |  |  |  |  |  |  |  |  |  |  |  |
| Daytime session (08:30–17:15) | ref |  |  |  | ref |  |  |  | ref |  |  |
| Evening session (17:15–20:00) | 1.62 | 0.76–3.46 | 0.214 |  | 1.27 | 0.72–2.22 | 0.409 |  | **1.43** | **1.17–1.74** | **<0.001** |
| Nighttime session (20:00–08:30) | 2.07 | 0.56–7.60 | 0.273 |  | **2.29** | **1.03–5.10** | **0.043** |  | **2.85** | **2.03–4.00** | **<0.001** |
| *Number of symptoms^d^* |  |  |  |  |  |  |  |  |  |  |  |
| None | ref |  |  |  | ref |  |  |  | ref |  |  |
| One | **2.20** | **1.31–3.67** | **0.003** |  | **1.93** | **1.23–3.04** | **0.004** |  | **2.10** | **1.47–2.99** | **<0.001** |
| Two | **3.91** | **2.34–6.51** | **<0.001** |  | **2.84** | **1.80–4.47** | **<0.001** |  | **3.02** | **2.12–4.31** | **<0.001** |
| Three or more | **7.08** | **4.08–12.31** | **<0.001** |  | **4.89** | **3.03–7.89** | **<0.001** |  | **5.64** | **3.88–8.18** | **<0.001** |
| *Consultation* |  |  |  |  |  |  |  |  |  |  |  |
| Medical consultation on physical symptoms | **1.76** | **1.02–3.03** | **0.043** |  | **2.50** | **1.55–4.03** | **<0.001** |  | **2.33** | **1.61–3.36** | **<0.001** |
| Request for involvement of a medical doctor | 4.64 | 0.47–45.77 | 0.189 |  | **5.90** | **1.58–22.07** | **0.008** |  | **7.18** | **2.44–21.08** | **<0.001** |
| Concerns and consultations on one's own life | 1.91 | 0.42–8.75 | 0.406 |  | 1.03 | 0.39–2.70 | 0.953 |  | 1.77 | 0.85–3.67 | 0.125 |
| Concerns and consultations on nearby people (e.g., family members, close contacts) | 2.63 | 0.92–7.54 | 0.071 |  | 1.92 | 0.77–4.81 | 0.164 |  | **5.36** | **2.66–10.80** | **<0.001** |

Abbreviations: aOR: adjusted odds ratio, 95%CI: 95% confidence interval

a, Incidence groups were defined using tertiles of the 7-day moving average.

b, aOR > 1: session lasting ≥ 15 minutes.

c, Binary logistic regression

d, Symptoms included fever (≥ 37.5°C), oxygen saturation ≤ 95%, respiratory symptoms (e.g., dyspnea, cough, sore throat), fatigue, digestive symptoms (e.g., vomiting, diarrhea), loss of smell or taste, poor diet or fluid intake, stress-related symptoms, need for emergency mental care, and other symptoms

**S1B Table. Factors associated with long telephone observation and consultation sessions under four alternative incidence-group definitions (the 25th and 75th percentiles of raw daily case counts)**

|  | **Low group^a^**  **(77.0–298.0 new cases/day)** | | |  | **Middle group^a^**  **(304.0–2967.0 new cases/day)** | | |  | **High group^a^**  **(3008.0–8085.0 new cases/day)** | | |
| --- | --- | --- | --- | --- | --- | --- | --- | --- | --- | --- | --- |
|  | **aOR^b^** | **95%CI** | ***P-*value^c^** |  | **aOR^b^** | **95%CI** | ***P-*value^c^** |  | **aOR^b^** | **95%CI** | ***P-*value^c^** |
| *Age (years)* |  |  |  |  |  |  |  |  |  |  |  |
| 65–79 | ref |  |  |  | ref |  |  |  | ref |  |  |
| 0–64 | 0.58 | 0.24–1.38 | 0.220 |  | 0.91 | 0.65–1.28 | 0.585 |  | 0.82 | 0.57–1.19 | 0.303 |
| ≥ 80 | **1.45** | **1.08–1.96** | **0.014** |  | **1.65** | **1.39–1.96** | **<0.001** |  | **1.75** | **1.46–2.09** | **<0.001** |
| *Sex/pregnancy category* |  |  |  |  |  |  |  |  |  |  |  |
| Female (non-pregnant) | ref |  |  |  | ref |  |  |  | ref |  |  |
| Male | 0.95 | 0.71–1.27 | 0.731 |  | 1.00 | 0.85–1.17 | 0.975 |  | 0.88 | 0.74–1.04 | 0.144 |
| Female (pregnant) | 0.21 | 0.02–1.83 | 0.156 |  | **0.50** | **0.25–0.97** | **0.041** |  | **0.30** | **0.13–0.66** | **0.003** |
| *Contact time* |  |  |  |  |  |  |  |  |  |  |  |
| Daytime session (08:30–17:15) | ref |  |  |  | ref |  |  |  | ref |  |  |
| Evening session (17:15–20:00) | 1.24 | 0.50–3.04 | 0.642 |  | 1.10 | 0.78–1.55 | 0.579 |  | **1.34** | **1.08–1.66** | **0.008** |
| Nighttime session (20:00–08:30) | 1.58 | 0.25–9.93 | 0.628 |  | **2.40** | **1.35–4.25** | **0.003** |  | **2.61** | **1.81–3.76** | **<0.001** |
| *Number of symptoms^d^* |  |  |  |  |  |  |  |  |  |  |  |
| None | ref |  |  |  | ref |  |  |  | ref |  |  |
| One | **1.82** | **1.01–3.27** | **0.045** |  | **1.99** | **1.37–2.89** | **<0.001** |  | **2.23** | **1.52–3.29** | **<0.001** |
| Two | **3.06** | **1.71–5.49** | **<0.001** |  | **3.13** | **2.16–4.53** | **<0.001** |  | **3.16** | **2.14–4.68** | **<0.001** |
| Three or more | **5.03** | **2.68–9.44** | **<0.001** |  | **5.90** | **4.00–8.71** | **<0.001** |  | **5.64** | **3.74–8.50** | **<0.001** |
| *Consultation* |  |  |  |  |  |  |  |  |  |  |  |
| Medical consultation on physical symptoms | **2.55** | **1.33–4.88** | **0.005** |  | **2.36** | **1.61–3.48** | **<0.001** |  | **2.26** | **1.51–3.37** | **<0.001** |
| Request for involvement of a medical doctor | 6.20 | 0.59–65.33 | 0.129 |  | **6.63** | **2.03–21.68** | **0.002** |  | **6.36** | **1.99–20.35** | **0.002** |
| Concerns and consultations on one's own life | 4.38 | 0.81–23.64 | 0.086 |  | 0.94 | 0.43–2.06 | 0.872 |  | 1.89 | 0.80–4.48 | 0.147 |
| Concerns and consultations on nearby people (e.g., family members, close contacts) | 2.59 | 0.80–8.44 | 0.114 |  | **2.71** | **1.27–5.78** | **0.010** |  | **5.51** | **2.60–11.69** | **<0.001** |

Abbreviations: aOR: adjusted odds ratio, 95%CI: 95% confidence interval

a, Incidence groups were defined using the 25th and 75th percentiles of raw daily case counts.

b, aOR > 1: session lasting ≥ 15 minutes.

c, Binary logistic regression

d, Symptoms included fever (≥ 37.5°C), oxygen saturation ≤ 95%, respiratory symptoms (e.g., dyspnea, cough, sore throat), fatigue, digestive symptoms (e.g., vomiting, diarrhea), loss of smell or taste, poor diet or fluid intake, stress-related symptoms, need for emergency mental care, and other symptoms

**S1C Table. Factors associated with long telephone observation and consultation sessions under four alternative incidence-group definitions (the 25th and 75th percentiles of the 3-day moving average)**

|  | **Low group^a^**  **(183.7–293.0 new cases/day)** | | |  | **Middle group^a^**  **(295.3–3049.0 new cases/day)** | | |  | **High group^a^**  **(3118.7–8064.7 new cases/day)** | | |
| --- | --- | --- | --- | --- | --- | --- | --- | --- | --- | --- | --- |
|  | **aOR^b^** | **95%CI** | ***P-*value^c^** |  | **aOR^b^** | **95%CI** | ***P-*value^c^** |  | **aOR^b^** | **95%CI** | ***P-*value^c^** |
| *Age (years)* |  |  |  |  |  |  |  |  |  |  |  |
| 65–79 | ref |  |  |  | ref |  |  |  | ref |  |  |
| 0–64 | 0.78 | 0.36–1.68 | 0.526 |  | 0.78 | 0.54–1.13 | 0.196 |  | 0.92 | 0.65–1.31 | 0.652 |
| ≥ 80 | **1.58** | **1.18–2.13** | **0.002** |  | **1.73** | **1.45–2.07** | **<0.001** |  | **1.67** | **1.40–1.99** | **<0.001** |
| *Sex/pregnancy category* |  |  |  |  |  |  |  |  |  |  |  |
| Female (non-pregnant) | ref |  |  |  | ref |  |  |  | ref |  |  |
| Male | 1.08 | 0.81–1.42 | 0.605 |  | 0.93 | 0.79–1.10 | 0.401 |  | 0.91 | 0.77–1.08 | 0.268 |
| Female (pregnant) | **0.10** | **0.01–0.87** | **0.037** |  | **0.48** | **0.23–0.99** | **0.047** |  | **0.35** | **0.17–0.73** | **0.005** |
| *Contact time* |  |  |  |  |  |  |  |  |  |  |  |
| Daytime session (08:30–17:15) | ref |  |  |  | ref |  |  |  | ref |  |  |
| Evening session (17:15–20:00) | 1.06 | 0.40–2.78 | 0.918 |  | 1.12 | 0.74–1.70 | 0.597 |  | **1.37** | **1.12–1.69** | **0.002** |
| Nighttime session (20:00–08:30) | 3.32 | 0.83–13.36 | 0.091 |  | **2.01** | **1.01–4.01** | **0.048** |  | **2.73** | **1.92–3.87** | **<0.001** |
| *Number of symptoms^d^* |  |  |  |  |  |  |  |  |  |  |  |
| None | ref |  |  |  | ref |  |  |  | ref |  |  |
| One | **2.29** | **1.24–4.23** | **0.008** |  | **1.81** | **1.26–2.62** | **0.001** |  | **2.28** | **1.54–3.38** | **<0.001** |
| Two | **4.56** | **2.49–8.34** | **<0.001** |  | **2.52** | **1.74–3.65** | **<0.001** |  | **3.42** | **2.31–5.06** | **<0.001** |
| Three or more | **9.06** | **4.73–17.35** | **<0.001** |  | **4.62** | **3.13–6.82** | **<0.001** |  | **5.95** | **3.94–8.98** | **<0.001** |
| *Consultation* |  |  |  |  |  |  |  |  |  |  |  |
| Medical consultation on physical symptoms | **1.51** | **0.79–2.88** | **0.211** |  | **2.33** | **1.59–3.41** | **<0.001** |  | **2.67** | **1.76–4.04** | **<0.001** |
| Request for involvement of a medical doctor | 4.68 | 0.48–46.02 | 0.185 |  | **4.72** | **1.44–15.47** | **0.010** |  | **8.85** | **2.75–28.46** | **<0.001** |
| Concerns and consultations on one's own life | 3.97 | 0.71–22.21 | 0.117 |  | 1.10 | 0.50–2.40 | 0.820 |  | 1.70 | 0.72–4.04 | 0.228 |
| Concerns and consultations on nearby people (e.g., family members, close contacts) | 2.11 | 0.63–7.05 | 0.223 |  | **2.54** | **1.17–5.52** | **0.019** |  | **6.00** | **2.85–12.67** | **<0.001** |

Abbreviations: aOR: adjusted odds ratio, 95%CI: 95% confidence interval

a, Incidence groups were defined using the 25th and 75th percentiles of the 3-day moving average.

b, aOR > 1: session lasting ≥ 15 minutes.

c, Binary logistic regression

d, Symptoms included fever (≥ 37.5°C), oxygen saturation ≤ 95%, respiratory symptoms (e.g., dyspnea, cough, sore throat), fatigue, digestive symptoms (e.g., vomiting, diarrhea), loss of smell or taste, poor diet or fluid intake, stress-related symptoms, need for emergency mental care, and other symptoms

**S1D Table.** **Factors associated with long telephone observation and consultation sessions under four alternative incidence-group definitions (the 25th and 75th percentiles of the 10-day moving average)**

|  | **Low group^a^**  **(227.7–277.6 new cases/day)** | | |  | **Middle group^a^**  **(279.9–3108.3 new cases/day)** | | |  | **High group^a^**  **(3221.2–5751.5 new cases/day)** | | |
| --- | --- | --- | --- | --- | --- | --- | --- | --- | --- | --- | --- |
|  | **aOR^b^** | **95%CI** | ***P-*value^c^** |  | **aOR^b^** | **95%CI** | ***P-*value^c^** |  | **aOR^b^** | **95%CI** | ***P-*value^c^** |
| *Age (years)* |  |  |  |  |  |  |  |  |  |  |  |
| 65–79 | ref |  |  |  | ref |  |  |  | ref |  |  |
| 0–64 | 0.70 | 0.34–1.46 | 0.342 |  | 0.77 | 0.54–1.11 | 0.167 |  | 0.94 | 0.66–1.35 | 0.736 |
| ≥ 80 | **1.41** | **1.04–1.90** | **0.025** |  | **1.74** | **1.46–2.08** | **<0.001** |  | **1.68** | **1.41–2.01** | **<0.001** |
| *Sex/pregnancy category* |  |  |  |  |  |  |  |  |  |  |  |
| Female (non-pregnant) | ref |  |  |  | ref |  |  |  | ref |  |  |
| Male | 0.97 | 0.73–1.29 | 0.857 |  | 0.97 | 0.82–1.15 | 0.758 |  | 0.90 | 0.76–1.07 | 0.216 |
| Female (pregnant) | 0.31 | 0.06–1.59 | 0.160 |  | **0.34** | **0.15–0.79** | **0.012** |  | **0.38** | **0.19–0.76** | **0.007** |
| *Contact time* |  |  |  |  |  |  |  |  |  |  |  |
| Daytime session (08:30–17:15) | ref |  |  |  | ref |  |  |  | ref |  |  |
| Evening session (17:15–20:00) | 1.71 | 0.68–4.33 | 0.255 |  | 0.97 | 0.63–1.49 | 0.896 |  | **1.39** | **1.13–1.71** | **0.002** |
| Nighttime session (20:00–08:30) | **4.98** | **1.21–20.43** | **0.026** |  | 1.67 | 0.82–3.40 | 0.161 |  | **2.72** | **1.91–3.87** | **<0.001** |
| *Number of symptoms^d^* |  |  |  |  |  |  |  |  |  |  |  |
| None | ref |  |  |  | ref |  |  |  | ref |  |  |
| One | **2.16** | **1.15–4.05** | **0.017** |  | **1.97** | **1.36–2.87** | **<0.001** |  | **2.18** | **1.48–3.21** | **<0.001** |
| Two | **4.11** | **2.21–7.64** | **<0.001** |  | **2.75** | **1.89–4.01** | **<0.001** |  | **3.20** | **2.17–4.73** | **<0.001** |
| Three or more | **6.99** | **3.59–13.61** | **<0.001** |  | **5.09** | **3.43–7.55** | **<0.001** |  | **5.75** | **3.82–8.65** | **<0.001** |
| *Consultation* |  |  |  |  |  |  |  |  |  |  |  |
| Medical consultation on physical symptoms | **1.97** | **1.00–3.87** | **0.049** |  | **2.22** | **1.50–3.27** | **<0.001** |  | **2.56** | **1.71–3.85** | **<0.001** |
| Request for involvement of a medical doctor | 3.61 | 0.33–38.90 | 0.291 |  | **5.88** | **1.74–19.90** | **0.004** |  | **6.81** | **2.11–21.95** | **0.001** |
| Concerns and consultations on one's own life | 0.81 | 0.07–8.86 | 0.863 |  | 1.00 | 0.45–2.21 | 0.994 |  | **2.46** | **1.07–5.67** | **0.034** |
| Concerns and consultations on nearby people (e.g., family members, close contacts) | 2.65 | 0.74–9.44 | 0.132 |  | **2.56** | **1.18–5.59** | **0.018** |  | **6.26** | **2.95–13.29** | **<0.001** |

Abbreviations: aOR: adjusted odds ratio, 95%CI: 95% confidence interval

a, Incidence groups were defined using the 25th and 75th percentiles of the 10-day moving average.

b, aOR > 1: session lasting ≥ 15 minutes.

c, Binary logistic regression

d, Symptoms included fever (≥ 37.5°C), oxygen saturation **≤** 95%, respiratory symptoms (e.g., dyspnea, cough, sore throat), fatigue, digestive symptoms (e.g., vomiting, diarrhea), loss of smell or taste, poor diet or fluid intake, stress-related symptoms, need for emergency mental care, and other symptoms
