# Supplementary material for: Factors associated with the duration of telephone observation and consultation sessions provided by the Hiroshima Prefecture Follow-up Center in the later stages of the COVID-19 pandemic in Japan
Source: PLoS One. 2026 Jun 26;21(6):e0352251. doi: 10.1371/journal.pone.0352251 (PMC13308847; doi:10.1371/journal.pone.0352251)
Supplement: S2 Table — (DOCX) [file pone.0352251.s002.docx]

**S2 Table. Ordinal logistic regression analysis of telephone observation and consultation duration (<15, 15–30, and ≥30 minutes)**

|  | **Low group^a^**  **(200.7–281.3 new cases/day)** | | |  | **Middle group^a^**  **(281.4–2975.9 new cases/day)** | | |  | **High group^a^**  **(3036.0–6248.0 new cases/day)** | | |
| --- | --- | --- | --- | --- | --- | --- | --- | --- | --- | --- | --- |
|  | **aOR^b^** | **95%CI** | ***P-*value^c^** |  | **aOR^b^** | **95%CI** | ***P-*value^c^** |  | **aOR^b^** | **95%CI** | ***P-*value^c^** |
| *Age (years)* |  |  |  |  |  |  |  |  |  |  |  |
| 65–79 | ref |  |  |  | ref |  |  |  | ref |  |  |
| 0–64 | 0.51 | 0.22–1.17 | 0.112 |  | 0.82 | 0.58–1.18 | 0.293 |  | 0.95 | 0.67–1.35 | 0.767 |
| ≥ 80 | **1.35** | **1.01–1.80** | **0.046** |  | **1.80** | **1.52–2.15** | **<0.001** |  | **1.67** | **1.40–1.99** | **<0.001** |
| *Sex/pregnancy category* |  |  |  |  |  |  |  |  |  |  |  |
| Female (non-pregnant) | ref |  |  |  | ref |  |  |  | ref |  |  |
| Male | 0.97 | 0.73–1.28 | 0.825 |  | 0.99 | 0.84–1.17 | 0.903 |  | 0.89 | 0.75–1.06 | 0.185 |
| Female (pregnant) | 0.38 | 0.07–2.01 | 0.253 |  | **0.36** | **0.16–0.80** | **0.012** |  | **0.33** | **0.16–0.69** | **0.003** |
| *Contact time* |  |  |  |  |  |  |  |  |  |  |  |
| Daytime session (08:30–17:15) | ref |  |  |  | ref |  |  |  | ref |  |  |
| Evening session (17:15–20:00) | 1.20 | 0.49–2.95 | 0.692 |  | 1.04 | 0.69–1.59 | 0.842 |  | **1.36** | **1.11–1.67** | **0.003** |
| Nighttime session (20:00–08:30) | 2.59 | 0.58–11.66 | 0.214 |  | **1.96** | **1.01–3.77** | **0.045** |  | **2.74** | **1.93–3.89** | **<0.001** |
| *Number of symptoms^d^* |  |  |  |  |  |  |  |  |  |  |  |
| None | ref |  |  |  | ref |  |  |  | ref |  |  |
| One | 1.75 | 0.99–3.09 | 0.054 |  | **1.95** | **1.34–2.82** | **<0.001** |  | **2.33** | **1.57–3.45** | **<0.001** |
| Two | **3.22** | **1.83–5.65** | **<0.001** |  | **2.84** | **1.95–4.12** | **<0.001** |  | **3.45** | **2.32–5.2** | **<0.001** |
| Three or more | **6.04** | **3.29–11.11** | **<0.001** |  | **5.09** | **3.43–7.55** | **<0.001** |  | **6.05** | **3.99–9.16** | **<0.001** |
| *Consultation* |  |  |  |  |  |  |  |  |  |  |  |
| Medical consultation on physical symptoms | **2.23** | **1.20–4.15** | **0.011** |  | **2.13** | **1.45–3.14** | **<0.001** |  | **2.57** | **1.70–3.90** | **<0.001** |
| Request for involvement of a medical doctor | 1.44 | 0.17–12.53 | 0.739 |  | **4.95** | **1.81–13.53** | **0.002** |  | **9.12** | **3.02–27.47** | **<0.001** |
| Concerns and consultations on one's own life | 4.00 | 0.74–21.59 | 0.107 |  | 0.93 | 0.41–2.08 | 0.853 |  | 1.99 | 0.86–4.61 | 0.109 |
| Concerns and consultations on nearby people (e.g., family members, close contacts) | **4.77** | **1.44–15.79** | **0.010** |  | 2.12 | 0.98–4.60 | 0.057 |  | **5.85** | **2.78–12.33** | **<0.001** |

Abbreviations: aOR: adjusted odds ratio, 95%CI: 95% confidence interval

a, Incidence groups were defined using the 25th and 75th percentiles of the 7-day moving average.

b, aOR > 1: 1 indicates higher odds of being in a longer call-duration category.

c, Ordinal logistic regression

d, Symptoms included fever (≥ 37.5°C), oxygen saturation ≤ 95%, respiratory symptoms (e.g., dyspnea, cough, sore throat), fatigue, digestive symptoms (e.g., vomiting, diarrhea), loss of smell or taste, poor diet or fluid intake, stress-related symptoms, need for emergency mental care, and other symptoms
