# Supplementary material for: Factors associated with the duration of telephone observation and consultation sessions provided by the Hiroshima Prefecture Follow-up Center in the later stages of the COVID-19 pandemic in Japan
Source: PLoS One. 2026 Jun 26;21(6):e0352251. doi: 10.1371/journal.pone.0352251 (PMC13308847; doi:10.1371/journal.pone.0352251)
Supplement: S3 Table — (DOCX) [file pone.0352251.s003.docx]

**S3 Table. Initial contact route/workflow and outcomes of medical coordination after nurse response by call duration**

|  | < 15 minutes | |  | ≥ 15 minutes | |
| --- | --- | --- | --- | --- | --- |
|  | **N** | **%** |  | **N** | **%** |
| *Initial contact route/workflow* |  |  |  |  |  |
| End-of-isolation contact | 10,702 | 98.0 |  | 218 | 2.0 |
| My HER-SYS-related contact | 712 | 92.2 |  | 60 | 7.8 |
| Initial contact from the Follow-Up Center | 4,774 | 86.3 |  | 755 | 13.7 |
| Direct call from a patient or a family member | 212 | 77.4 |  | 62 | 22.6 |
| Others | 4,504 | 90.2 |  | 491 | 9.8 |
| *Outcome of medical coordination after nurse response* |  |  |  |  |  |
| End-of-isolation contact | 10,331 | 98.1 |  | 198 | 1.9 |
| Hospitalized | 20 | 80.0 |  | 5 | 20.0 |
| Coordinated for hospitalization but could not be admitted | 0 | – |  | 0 | – |
| Transferred response from the follow-up center to a PHC | 16 | 80.0 |  | 4 | 20.0 |
| Ongoing (coordinating for hospitalization, contacting a PHC) | 20 | 60.6 |  | 13 | 39.4 |
| Others | 443 | 92.1 |  | 38 | 7.9 |
| This table is based on the descriptive analysis dataset, which included records with non-missing incidence-group classification and call-duration outcome (n = 22,490). Initial contact route/workflow categories were mutually exclusive and summed to the descriptive analysis dataset. For outcomes of medical coordination after nurse response, only sessions with a corresponding coordination outcome are shown, and the denominator for each row was the number of sessions with that outcome; therefore, these numbers do not sum to the total. | | | | | |
