# Supplementary material for: Factors associated with the duration of telephone observation and consultation sessions provided by the Hiroshima Prefecture Follow-up Center in the later stages of the COVID-19 pandemic in Japan
Source: PLoS One. 2026 Jun 26;21(6):e0352251. doi: 10.1371/journal.pone.0352251 (PMC13308847; doi:10.1371/journal.pone.0352251)
Supplement: S4 Table — (DOCX) [file pone.0352251.s004.docx]

**S4.** **Distribution of individual symptoms by symptom-count category**

|  | **One** | **Two** | **Three or more** |
| --- | --- | --- | --- |
| Respiratory symptoms (e.g., dyspnea, cough, sore throat) | 3,319 (67.6%) | 3,620 (90.5%) | 1,576 (94.5%) |
| Other symptoms | 921 (18.8%) | 2,337 (58.5%) | 1,246 (74.7%) |
| Oxygen saturation ≤ 95% | 343 (7.0%) | 586 (14.7%) | 401 (24.1%) |
| Fever (≥ 37.5°C) | 174 (3.5%) | 693 (17.3%) | 798 (47.9%) |
| Fatigue | 61 (1.2%) | 405 (10.1%) | 775 (46.5%) |
| Digestive symptoms (e.g., vomiting, diarrhea) | 52 (1.1%) | 158 (4.0%) | 258 (15.5%) |
| Loss of smell or taste | 23 (0.5%) | 113 (2.8%) | 166 (10.0%) |
| Poor diet or fluid intake | 15 (0.3%) | 77 (1.9%) | 170 (10.2%) |
| Stress-related symptoms | 0 (0%) | 5 (0.1%) | 4 (0.2%) |
| Need for emergency mental care | 0 (0%) | 2 (0.1%) | 2 (0.1%) |

This table is based on the regression dataset after excluding records with missing covariates (n = 22,372). Therefore, the denominators differ slightly from those in the descriptive analysis based on records with non-missing incidence-group classification and call-duration outcome (n = 22,490).
